# Asc1 Supports Cell-Wall Integrity Near Bud Sites by a Pkc1 Independent Mechanism

**DOI:** 10.1371/journal.pone.0011389

**Published:** 2010-06-30

**Authors:** Daniel Melamed, Lavi Bar-Ziv, Yossi Truzman, Yoav Arava

**Affiliations:** Department of Biology, Technion – Israel Institute of Technology, Haifa, Israel; University of Washington, United States of America

## Abstract

**Background:**

The yeast ribosomal protein Asc1 is a WD-protein family member. Its mammalian ortholog, RACK1 was initially discovered as a receptor for activated protein C kinase (PKC) that functions to maintain the active conformation of PKC and to support its movement to target sites. In the budding yeast though, a connection between Asc1p and the PKC signaling pathway has never been reported.

**Methodology/Principal Findings:**

In the present study we found that *asc1*-deletion mutant (*asc1*Δ) presents some of the hallmarks of PKC signaling mutants. These include an increased sensitivity to staurosporine, a specific Pkc1p inhibitor, and susceptibility to cell-wall perturbing treatments such as hypotonic- and heat shock conditions and zymolase treatment. Microscopic analysis of *asc1*Δ cells revealed cell-wall invaginations near bud sites after exposure to hypotonic conditions, and the dynamic of cells' survival after this stress further supports the involvement of Asc1p in maintaining the cell-wall integrity during the mid-to late stages of bud formation. Genetic interactions between *asc1* and *pkc1* reveal synergistic sensitivities of a double-knock out mutant (*asc1*Δ/*pkc1*Δ) to cell-wall stress conditions, and high basal level of PKC signaling in *asc1*Δ. Furthermore, Asc1p has no effect on the cellular distribution or redistribution of Pkc1p at optimal or at cell-wall stress conditions.

**Conclusions/Significance:**

Taken together, our data support the idea that unlike its mammalian orthologs, Asc1p acts remotely from Pkc1p, to regulate the integrity of the cell-wall. We speculate that its role is exerted through translation regulation of bud-site related mRNAs during cells' growth.

## Introduction

Asc1p is a member of the WD-40 repeat protein family that adopts a seven-bladed β-propeller structure [1], [2]. It was also observed to be a genuine ribosomal protein [1]–[5], with this function conserved from yeast to human. A cryo-EM study mapped Asc1, as well as its human ortholog, RACK1, to the small ribosomal subunit head region near the mRNA exit tunnel [1].

RACK1 was initially cloned from rat brain cDNA library as an intracellular Receptor for Activated Protein C Kinase [6], with PKCβII being the preferred binding partner [7], [8], [9]. Interaction of PKC with RACK1 is thought to hold PKC in an active conformation and to target PKC to appropriate intracellular locations [8], [10]. Later studies positioned RACK1 at a central point for multiple cellular functions, as it was found to serve as a scaffold protein for many component from diverse signaling cascades [11]–[20], with some of them able to interact simultaneously with RACK1, and therefore allow it to integrate inputs from distinct signaling pathways [21].

Based on these observations, it was suggested that RACK1, as a part of the 40 S ribosomal subunit, serves as a docking site for signaling molecules that regulate the activity of translation initiation factors or recruit mRNA-binding proteins to the ribosome [22]. Indeed, it was shown that the mammalian PKCβII interacts with RACK1 while the last associates with translating ribosomes, and that this interaction leads to phosphorylation of the translation initiation factor eIF6, which induce translation initiation [23].

The ability of mammalian RACK1 to compete for localization to the yeast ribosome and to complement phenotypes of asc1 deletion mutant, suggest that these two proteins share similar functions [5]. However, in yeast, the contribution of Asc1p to mRNA translation is not clear. At optimal growth conditions Asc1p is not essential, suggesting that this ribosomal protein is dispensable for the general translation process. In addition, connections between Asc1p and signaling pathways were only recently established. Asc1p was shown to function as G-protein β subunit for the Gα- Gpa1 protein, which is a part of the glucose-stimulated cAMP/PKA signaling pathway, and to inhibit its guanine-nucleotide exchange activity that is required for glucose-signal transmission [24]. In addition, Asc1p was identified as a possible component in the mating pheromone MAPK signaling [25]. However, a connection between Asc1p and the PKC signaling pathway in *S.cerevisiae* was never reported.

In the present study, we investigated the relationship between Asc1p and the PKC signaling pathway by following the phenotypes of *asc1*-deletion mutant and its genetic interactions with *PKC1*. We point Asc1p as a factor required for the integrity of the cell-wall near the bud site, and suggest that this function is independent of Pkc1p.

## Materials and Methods

### Yeast strains, plasmids and growth conditions

The following *Saccharomyces cerevisiae* strains were used: BY4741 (MATa; *his3Δ1; leu2Δ0; met15Δ0; ura3Δ0*), *asc1*Δ (MATa, *his3*Δ*1 leu2*Δ*0 met15*Δ*0 ura3*Δ*0;* *asc1::kanMX4*)(Euroscarf). *pkc1*Δ (*MATa leu2–3 112 ura3–52 trpl-1 his4 can1r, pkc1Δ::LEU2*) and its isogenic wild-type, EG123 (MATa *leu2–3 112 ura3–52 trpl-1 his4 can1r*), were kindly provided by Dr. A. Tartakoff [26]. The double knock-out strain *asc1*Δ/*pkc1*Δ was constructed by sporulation of the mating products of *asc1*Δ and *pkc1*Δ strains, and selection for germinated spores on synthetic complete media with geneticin (G418) and without leucine. Correct construction of the strain was further verified by Northern blot analysis [27]. The plasmid PDL468 (pGAL-PKC1^wt^-HA, URA3, CEN) was kindly provided by Dr. J. Gray [28], The plasmids PDL469 (pGAL-PKC1^K853R^-HA URA3, CEN) and PBM743 (pGAL-PKC1^R398A^, URA3, CEN) were kindly provided by Dr. A. Tartakoff [26], and the plasmid PVD67 (pPKC1-PKC1-GFP, URA3, 2 µ) was kindly provided by Dr. M.S. Cyert [29].

Cells were grown at 30°C in YPD unless otherwise mentioned. Plasmids were maintained by growing the cells in appropriate selection media (Synthetic dropout with the necessary supplements). For hypotonic shock conditions cells were grown in the presence of NaCl, KCl, or Sorbitol at the specified concentrations, for about 24 hours to logarithmic growth phase (OD600 0.6–0.8), collected at 4000 rpm for 3 minutes at room temperature, and resuspended in YPD media with no supplemental osmolyte for the indicated time points.

### Measuring yeast sensitivity to staurosporine

Yeast were grown over-night to logarithmic phase in YPD or YPD supplemented with 0.8 M NaCl, and then diluted to ∼100,000 cells/ml (OD600 of 0.01), in 500 µl of the same medium. At the time specified in the Results section, each sample was divided into two microfuge tubes, each containing 96 µl of culture. The two cultures were supplemented with 4 µl of 1 mg/ml staurosporine (Sigma S3939)(final concetration 40 µg/ml), or with 4 µl of water. Growth was monitored throughout the experiment by counting the number of cells with hemocytometer.

### Microscopic imaging

To observe cells before and after hypotonic shock treatment, 5 µl of cells were fixed by 4% paraformaldehyde for 10 min and visualized by an Olympus BX61TRF motorized microscope, equipped with a DP70 digital camera, using a 40× objective. To follow PKC1-GFP localization, cells were fixed as described, and images were obtained by a Nikon Eclipse 50i microscope with a 100× immersion objective and recorded by a Nikon DS-5M camera. Images were processed digitally using Adobe Photoshop (Adobe Systems, Inc.).

### Zymolase treatment

Cells were grown in YPD plus 1 M NaCl for ∼24 hrs to mid-logarithmic phase and concentrated to an OD600 of 5.0 in 2 ml microfuge tube containing 1760 µl YPD supplemented with 1 M NaCl, 200 µl 1 M DTT and 40 µl of 10 mg/ml Zymolase (ImmunO). At the indicated time points, the turbidity of each sample was measured immediately after the addition of 100 µl aliquot from the reaction tube into a spectrophotometer cuvette already containing 800 µl media and 100 µl of 10% SDS (final conc. 1%), which rapidly lyse cells with severe cell-wall damages. To follow Pkc1-GFP re-localization upon cell-wall stress, exponentially growing yeast were cultured in the presence of 0.1 M DTT and 0.2 mg/ml Zymolase for one hour.

### Measuring effects of Pkc1p mutations on viability

Cells were grown to mid-logarithmic phase in medium lacking uracil and in the presence of 2% glucose at 25°C. Cells were then harvested by centrifugation at 4000 rpm for 4 min at room temperature, washed once with water and diluted in water to a final concentration of 10^4^ cells/ml. 100 µl of each sample (∼1000 cells) were seeded on plates lacking uracil and either containing 2% glucose (no induction) or containing 2% galactose and 0.2% sucrose (induction). Colony forming units (CFU) were counted after three days of incubation at 25°C, and the average ratio for glucose/galactose CFU was calculated from three independent repeats.

#### Cellular fractionation

Cellular fractionation was based on the protocol of Frey *et. al* [30] for ER-membrane enrichment. Cells were grown to mid-logarithmic phase in YPD media. Cycloheximide was added to a final concentration of 0.1 mg/ml, and cells were harvested (4000 rpm, 4 min, 4°C) and resuspended in 400 µl of ice-cold membrane-fractionation buffer (20 mM Hepes-KOH pH 7.6, 100 mM potassium acetate, 5 mM magnesium acetate, 2 mM dithiothreitol, 0.1 mg/ml cycloheximide and 0.1 mM PMSF) either with or without additional 20 mM EDTA. After the addition of 1 ml glass beads, cells were lysed by two rounds of vigorous vortexing for 90 seconds at 4°C with bead-beater. Recovered lysates were centrifuged for 2 min at 1,200× g to remove cell debris. The remaining crude lysate was fractionated by centrifugation at 6,000× g for 20 min at 4°C to cytosol- containing supernatant, and ER membrane- containing pellet [30]. Equivalent amounts of proteins from each fraction were subjected to Western blot analysis.

#### Western blot analysis

Western analysis was performed as previously described [31]. Anti-HA monoclonal antibody (Covance MMS-101P) was used at a 1∶4000 dilution. Rabbit anti-Asc1p, generously provided by Dr. A. Link [5] was used at 1∶5000 dilution. Monoclonal anti-Pab1p antibody was a gift from Dr. Mordechai Choder (Technion – Israel Institute of Technology), and was used at 1∶10000 dilution. Anti-Hexokinase-HRP conjugated antibody was used at 1∶50000 dilution and was kindly provided by Michael Glickman (Technion – Israel Institute of Technology). Anti-mouse IgG-HRP conjugated (Sigma A5906) and Anti-rabbit IgG-HRP conjugated (Sigma A9169), were used at 1∶10000 dilution.

## Results

### *asc1Δ* strain display phenotypic linkage with the PKC signaling pathway

**Figure 1:**
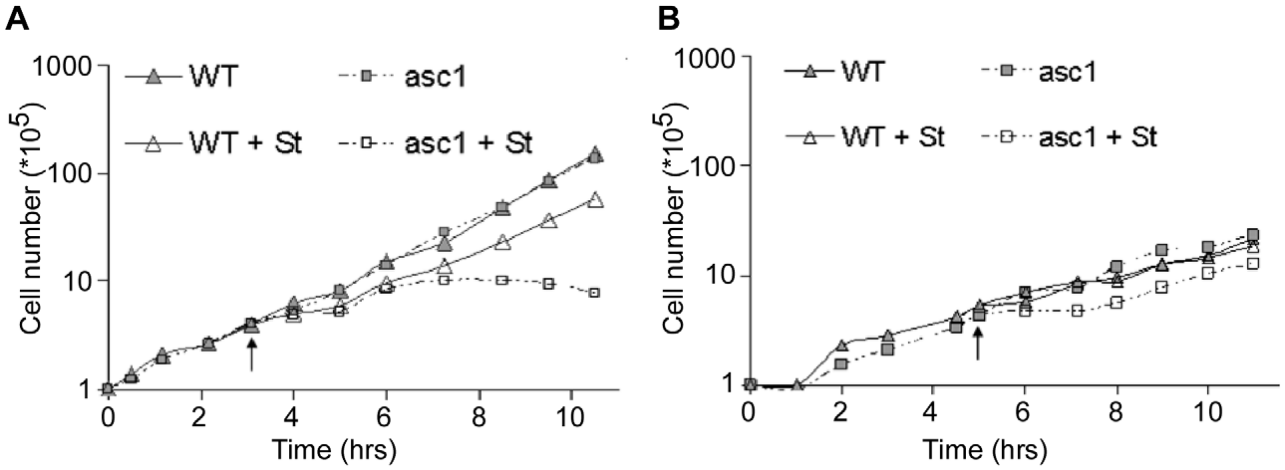
*asc1Δ* is sensitive to a PKC inhibitor. Wild-type and asc1Δ cells were grown to mid-logarithmic phase in rich media (YPD) in the absence (A) or in the presence of 0.8 M NaCl as an osmotic stabilizer (B), and diluted to concentrations of 10^5^ cells/ml. At the indicated time points (marked by black arrows) each culture was divided to two, and the two halves were supplemented either with staurosporine (+St) to a final concentration of 40 µg/ml or with an equal volume of water. Cell growth was monitored before and after Staurosporine addition by counting the cells' number using a hemocytometer.

The mammalian ortholog of Asc1p (RACK1) acts as a receptor for an activated PKCβ isoform [6]. In yeast, the PKC signaling pathway is activated in response to cell-wall stress conditions and controls cell-wall integrity at all stages of cell growth. Accordingly, mutations that interfere with this pathway result in hypersensitivity to various cell-wall perturbing treatments. It is therefore expected that if Asc1p function is directly or indirectly linked to PKC signaling, then loss of asc1 gene will share similar phenotypes with other mutations in the PKC pathway. To study this, we subjected an asc1-deletion mutant (*asc1*Δ) to staurosporine, a protein kinase inhibitor of which Pkc1p appears to be its primary target [32], [33]. Following addition of staurosporine (40 µg/ml) to exponentially growing yeast cultures, *asc1*Δ strain ceased to proliferate while its parental strain was almost unaffected by the drug (Fig. 1A). Significantly, this growth defect was suppressed by the addition of an osmotic stabilizer (0.8 M NaCl) to the media (Fig. 1B), linking the staurosporine sensitivity of *asc1*Δ to cell-wall defects resulting from loss of Pkc1p activity.

**Figure 2:**
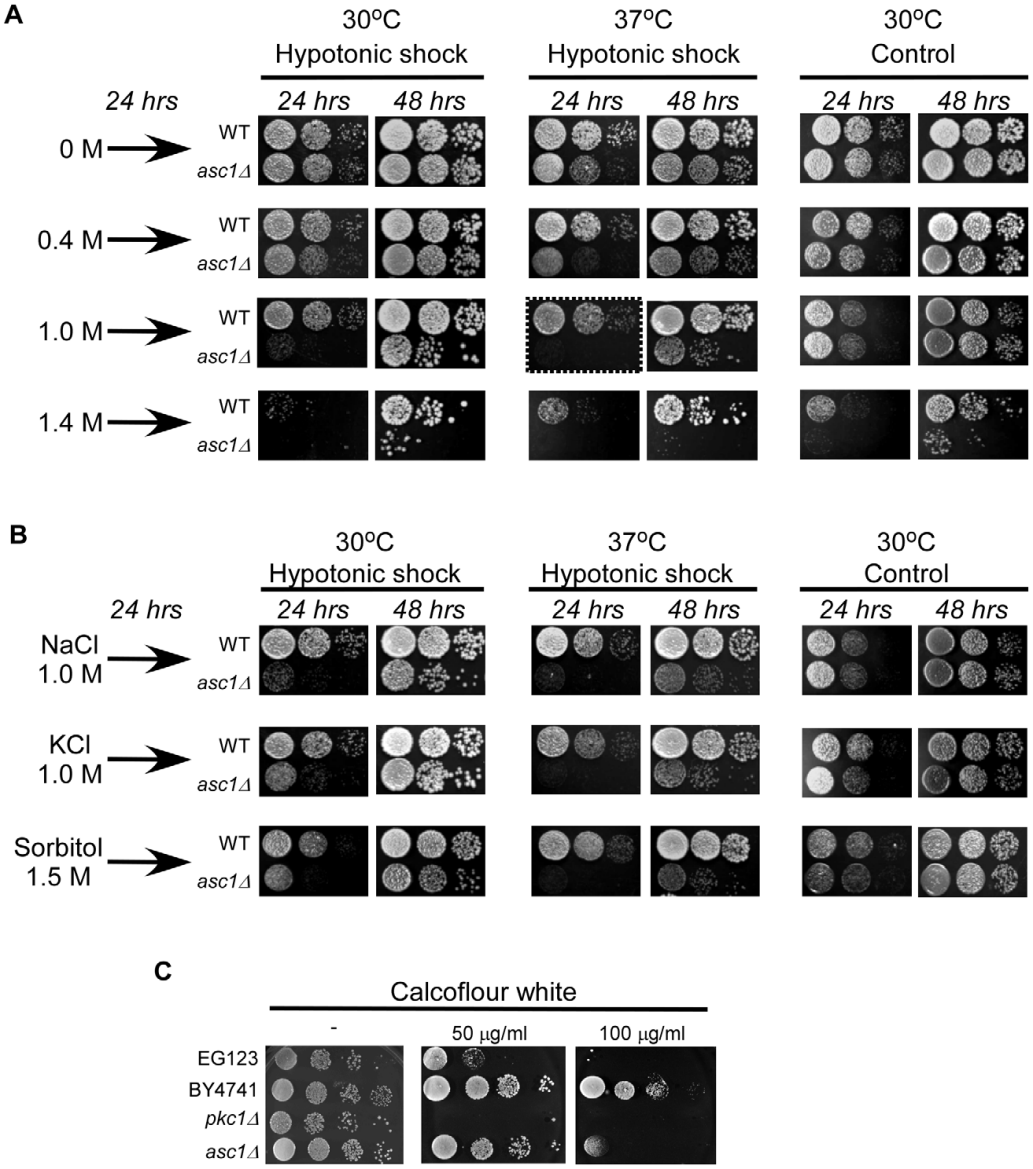
Response of *asc1Δ* cells to cell wall stresses. **The indicated** strains were grown for 24 hrs at 30°C in liquid YPD media containing 0 M, 0.4 M, 1 M or 1.4 M NaCl (A) or in the presence of 1 M NaCl, 1 M KCl, or 1.5 M Sorbitol (B), or 0.5 M NaCl (C). To impose hypotonic shock, cells were plated in a dilution series on YPD plates containing no supplemental osmolyte and grown either at 30°C (“30°C, Hypotonic shock”) or at 37°C (“37°C, Hypotonic shock”). For isotonic control, cells were spotted in a dilution series on YPD plates containing the same osmolyte concentrations as they grew in, and cultured at 30°C (“30°C Control”). Pictures of spotted colonies were taken after 24 hours (left panels) and 48 hours (right panels). To impose calcoflour white stress cells were plated on plates supplemented with 0.5 M NaCl and the indicated calcoflour white concentrations.

Additionally, we examined *asc1*Δ sensitivity to hypotonic stress and to elevated temperatures, two stimuli that damage the fluidity of the cell-wall and activate the PKC signal transduction pathway [34], [35]. In order to test the growth response to different strengths of hypotonic shock conditions, cells were grown at 30°C in rich media (YPD) containing different concentrations of NaCl (0, 0.4, 1 or 1.4 M) and after 24 hours serially diluted and seeded on YPD plates containing no NaCl (0 M) (Fig. 2A, left columns). The growth response to elevated temperatures was tested by growing the cells at 37°C (Fig. 2A, middle columns). Monitoring cell growth after 24 and 48 hours demonstrates that following exposure to extreme hypotonic shock conditions (from a 1 M NaCl starting point or above) or to elevated temperature, *asc1*Δ strain presents delayed resumption of growth and reduced survival, as compared to the wild type strain. The effect on growth was further enhanced when the two stress conditions were combined, with the strongest sensitivity when external osmolarity was reduced from 1 M to 0 M NaCl and growth temperature was 37°C (Fig. 2A dashed-line framed panel). To establish the generality of the sensitivity to hypotonic stress, the same experimental procedure was repeated using KCl and sorbitol (Fig. 2B). Indeed, increased sensitivities to hypotonic conditions and to combined heat shock were demonstrated after the cells were grown with either of the osmolytes, showing that this effect is not restricted to NaCl.

We have also examined the sensitivity of *asc1Δ* cells to calcoflour white (CFW), a cell-wall damaging agent (Fig. 2C). *asc1Δ* cells are much more sensitive to CFW than their parental strain (BY4741), as can clearly be seen on the plates supplemented with 100 µg/ml. As expected, cells deleted of Pkc1p are also sensitive to CFW and this can be observed already at a concentration of 50 µg/ml. Note that the parental strain of *pkc1Δ* (EG123) is much more sensitive to CFW than the parental strain of *asc1Δ* (BY4741), hence the difference is sensitivity between their progenies.

**Figure 3:**
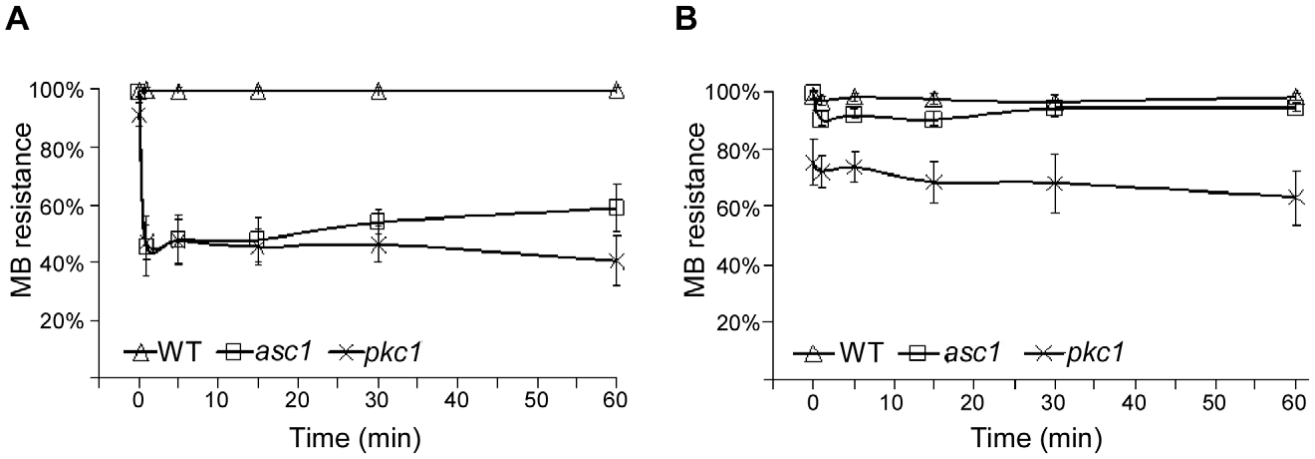
Hypotonicity cause rapid reduction in viability of dividing *asc1Δ* cells. Cells were grown in YPD supplemented with 1 M NaCl either to logarithmic growth phase (A) or to stationary phase (B). Cells were then shifted to media without NaCl to create hypotonic shock. At the indicated time points, dead cells were counted by Methylene Blue (MB) staining.

To follow the dynamics of the loss of viability upon hypotonic shock, we stained *asc1*Δ with methylene-blue, a dye that stain dead, or physically damaged cells [36], [37], at different time points after the cells were subjected to hypotonic shock. Interestingly, while the parental strain remained resistant to methylene-blue staining throughout the experiment, more than half of *asc1*Δ cells were stained within one minute following the shift to hypotonic conditions with no additional staining at the subsequent time points (Fig. 3A). This result indicates that *asc1*Δ loss of viability occurs immediately upon exposure to hypotonic treatment, which is consistent with abrupt cell-wall damage. This effect was extremely similar to the one observed for *PKC1*-deleted cells (*pkc1*Δ). Yet, the survival of the *pkc1*Δ strain showed also constant reduction in time (Fig. 3A) and eventually ended with a complete loss of viability (data not shown). When the hypotonic shock was exerted on cells at stationary growth phase, both *asc1*Δ and *pkc1*Δ were highly resistance to the immediate effect on viability (Fig. 3B). This shows that the two strains share similar vulnerability to hypotonic shock during exponential growth.

**Figure 4:**
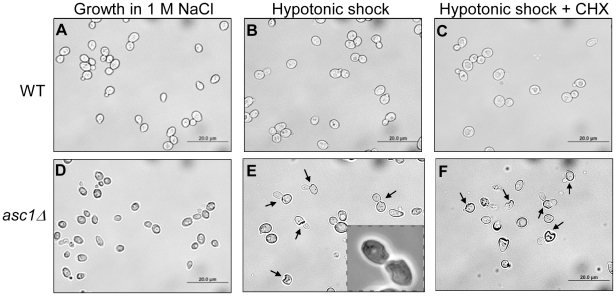
*asc1Δ* cells display aberrant cell-wall morphologies upon exposure to hypotonic conditions. Microscopic observations of wild-type and *asc1Δ* cells either under sustained growth in rich media (YPD) containing 1M NaCl (A, D), or following one hour exposure to hypotonic shock conditions (shift from 1 M to 0 M NaCl containing YPD media)(B, E), or in hypotonic conditions in the presence of 0.1 mg/ml Cycloheximide (CHX) (C, F). Arrows point to cell-wall deformations sites. Inset in E is a higher magnification of deformed cells.

Analysis of the cellular morphology of *asc1*-deletion strain before and after shifting the cells to hypotonic media demonstrated notable cell-wall invaginations that occurred mainly near the sites of the emerging buds (Fig. 4). Importantly, the aberrant cell-wall morphology was not linked to Asc1p effect on de-novo synthesis of proteins in response to the hypotonic shock, because arresting the translation process by Cycloheximide had no effect on the morphology of *asc1*Δ and its parental strain during the hypotonic stress (Fig. 4 compare C to B and F to E). Taken together, these results indicate that Asc1p role in cell-wall metabolism is concentrated mainly in budding sites. Unlike *asc1*Δ's response, *pkc1*Δ cells displayed severe deformations throughout the entire cell contour upon exposure to hypotonic stress conditions (data not shown). This comes in agreement with Pkc1p role in regulating and maintaining the integrity of the cell-wall at all steps of cell-cycle.

### Genetic interactions suggest that Asc1 and Pkc1 proteins do not act in concert

**Figure 5:**
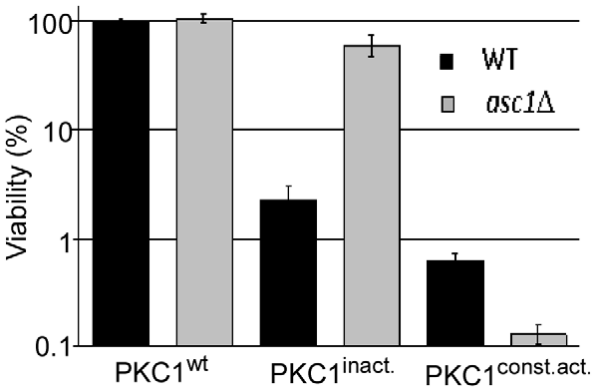
*asc1Δ* sensitivities to Pkc1 mutations. *asc1Δ* and its parental strain carrying plasmids that express either wild-type (wt), inactive (inact.) or constitutively-active (const.act.) forms of Pkc1p under galactose-inducible promoter were grown to mid logarithmic phase in non-inducing conditions (with glucose as a carbon source). The effect of each Pkc1p variant on survival was determined by seeding equal amounts of cells on plates containing either the inducing or the non-inducing carbon source, and calculating the induced/non-induced ratio for the colony forming units (CFU). All values were normalized to the wild-type Pkc1, which was set to 100%. Note that the Y-axis is in logarithmic scale.

To further establish the connection between Asc1p and the PKC signaling pathway we tested the effect of two variants of Pkc1 protein on the survival of *asc1*Δ cells. *asc1*Δ and its parental strain were transformed with plasmids that promote the expression of an inactive (Pkc1^K853R^) or a constitutive-active (Pkc1^R398A^) kinase mutants from a galactose-inducible promoter [32]. We determined the effect of the Pkc1p variants on the viability of each strain by comparing the number of colony forming units (CFU) on plates containing an inducing or non-inducing sugar source (Fig. 5). Over expressing the wild-type Pkc1p showed no effect on cell survival, in agreement with previous reports [28], [32]. However, over-expressing the inactive or the constitutive-active mutants affected the viability of *asc1*Δ and its parental strain differentially: *asc1*Δ cells were highly resistance to the deleterious effect of the inactive kinase mutation that was observed in the parental strain (∼60% *vs*. ∼2% viability, respectively), and showed increased sensitivity to the constitutive-active form of Pkc1p (∼0.15% *vs*. ∼0.6% viability in the parental strain). The simplest explanation for these observations is that *asc1*Δ cells have higher intrinsic activity of the PKC signaling pathway. The higher PKC activity therefore partially overcome the dominant-negative effect of the kinase-dead mutation and sum up with the constitutive-active mutant to produce a higher toxic effect.

**Figure 6:**
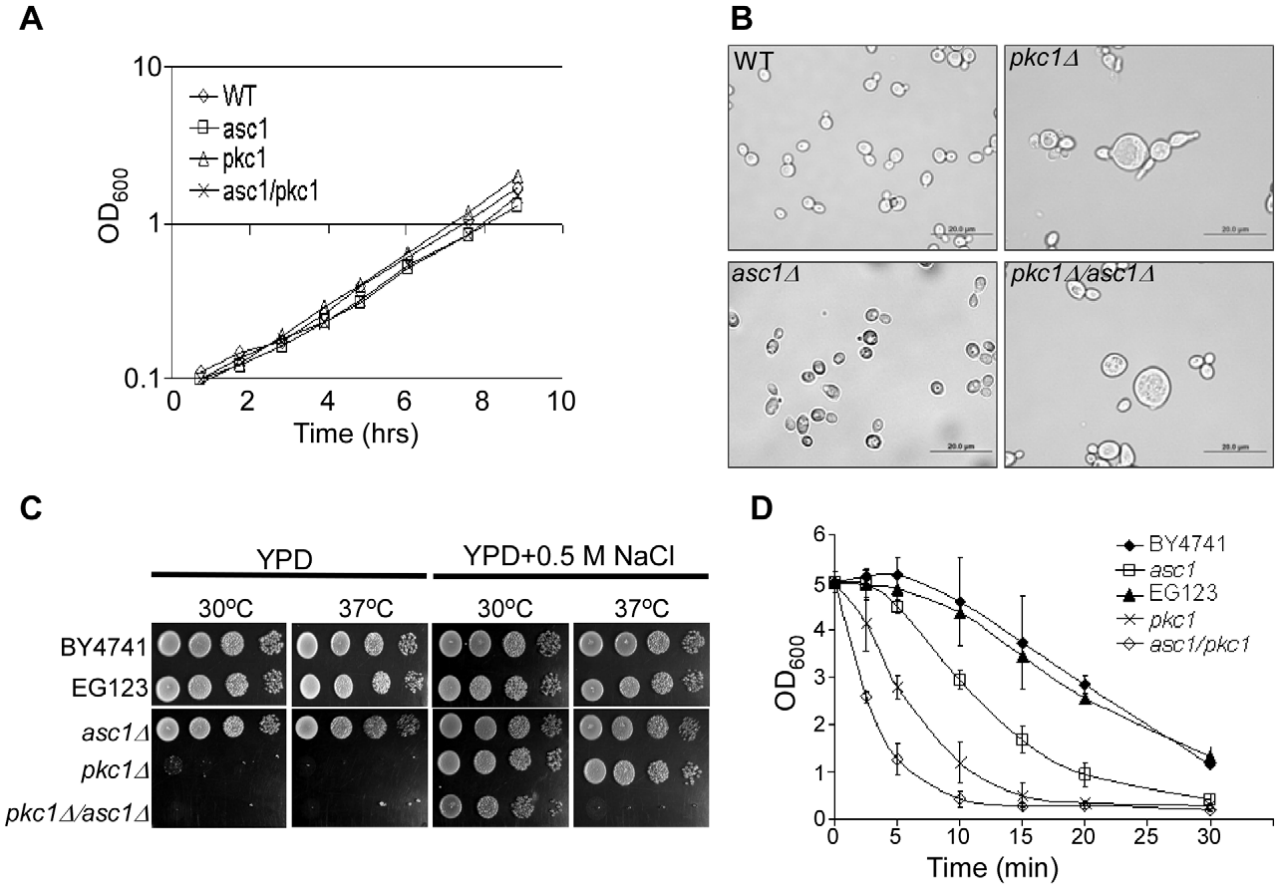
Phenotypes of *asc1Δ/pkc1Δ*. A) The indicated strains were grown in rich media in the presence of 0.5 M NaCl to logarithmic growth phase, and diluted to an OD_600_ of 0.1. Growth was monitored by measuring the OD_600_ values at the indicated time points. Results of one representative experiment out of three are shown. B) Microscopic morphology of the indicated strains when grown in rich media plus 1 M NaCl. C) Hypotonic shock sensitivity of the indicated strains. Cells were grown to mid-log phase in liquid YPD media supplemented with 0.5 M NaCl, serially diluted and plated on YPD plates with or without 0.5 M NaCl. Pictures were taken after 48 hrs of incubation at 30°C. C) Cell were grown in liquid YPD +0.5 M NaCl to mid-log phase, serially diluted and spotted on YPD plates either with no added NaCl, or with 0.5 M NaCl. Plates were incubated at 30°C or at 37°C. D) Sensitivity to Zymolase. Cells were grown in YPD +1 M NaCl to mid-logarithmic phase and concentrated to an OD_600_ of 5.0. Samples were then treated with 0.2 mg/ml Zymolase, and at the indicated time points cell-wall sensitivity was determined by adding SDS (final concentration 1%) and measuring the sample's turbidity.

We further constructed a double knockout haploid strain carrying deletions of both *ASC1* and *PKC1* genes (*asc1*Δ/*pkc1*Δ). This strain showed no considerable change in growth rate compared to single deletion mutants of *asc1* and *pkc1* (Fig. 6A) and presented some of the *pkc1Δ* strain phenotypic hallmarks, such as the existence of giant cells [38] (Fig. 6B), and the necessity for an osmotic stabilizer for growth [35] (Fig. 6C). Yet, it displayed synergistic sensitivities to some cell-wall perturbation treatments: It was unable to grow at elevated temperatures even in the presence of an osmotic stabilizer (Fig. 6C) and showed greater sensitivity to Zymolase (e.g. see the 2.5 min time point at Fig. 6D). This exaggerated sensitivity of the double knockout mutant may indicate that Asc1 and Pkc1 proteins act through different mechanisms to maintain the integrity of the cell-wall.

### Asc1p has no effect on Pkc1p localization

**Figure 7:**
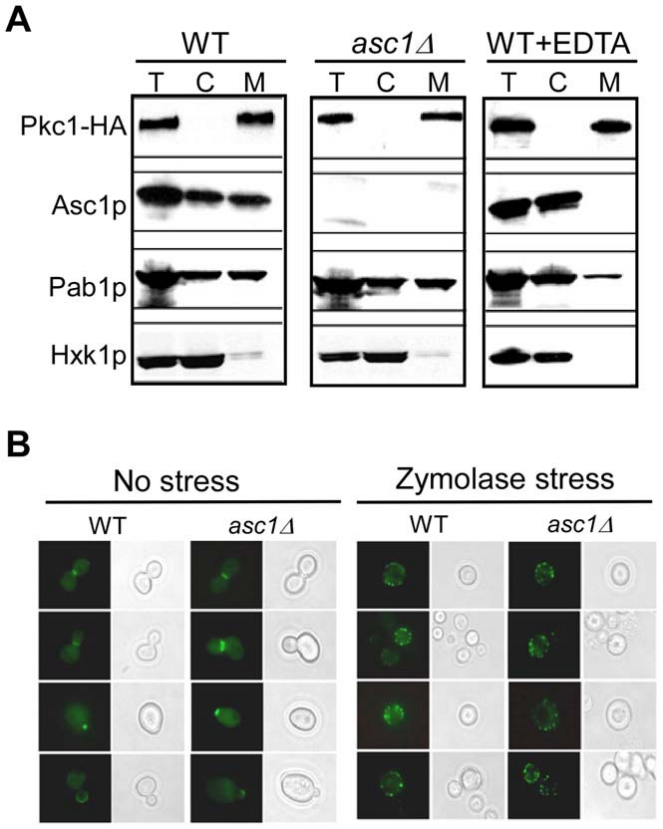
Asc1p has no effect on Pkc1p localization. A) Extracts of either wild-type (WT) or *asc1Δ* strains expressing HA-tagged Pkc1p were fractionated to membrane pellet (M) and cytosolic supernatant (C). The procedure for the WT strain was performed either in the absence or in the presence of 20 mM EDTA. Equivalent amounts of an unfractionated sample (T), cytosolic (C) and membranous (M) samples were subjected to Western analysis with antibodies recognizing the HA moiety of PKC1 or Asc1p. Pab1p (detected by α-pab1 antibody) was used as a marker for polysomes-associated factor, and Hxk1p (detected by α-Hxk1p antibody) was used as a cytosolic marker. B) Pkc1p-GFP was visualized in wild-type and *asc1Δ* cells grown to mid-log phase either without (“no stress”) or with one hour treatment with Zymolase (“Zymolase stress”).

One of the outcomes of PKCβII-RACK1 interaction in mammalian cells is the targeting of PKCβII to specific intracellular location, which vary between cell types [6], [8], [10]. To study whether Asc1p affects the cellular localization of Pkc1p, we fractionated lysates from wild-type and *asc1*Δ cells to their membranous and cytosolic parts [30] and tested the distribution of Asc1p and Pkc1p between the two compartments. Asc1p appeared to be evenly distributed between the membranous and the cytosolic fractions in wild-type cells (Fig. 7A), similarly as the polysome-associated factor Pab1p. Pkc1p however, appeared almost exclusively in the membranous part (Fig. 7A). This association is independent of Asc1p, as it was unaffected by *asc1*-gene deletion (Fig. 7A). Moreover, EDTA treatment, which leads to disassembly of polysomes and consequently to the release of Asc1p from the membrane part (Fig. 7A), did not change the membrane association of Pkc1p.

We also followed the localization of a Pkc1-GFP fusion protein that consists of the entire Pkc1p fused through its C-terminus to a green fluorescent protein. Expressing Pkc1-GFP from a high-copy plasmid in wild-type cells resulted in localization of this protein at the bud tip in small- to medium-sized buds and at the bud neck in large budded cells (Fig. 7B) as previously reported [29], [39]. This localization pattern was unaffected when the same fusion protein was expressed in *asc1*Δ cells (Fig. 7B). Additionally, when wild-type and *asc1*Δ cells were subjected to a Pkc1p-activating cell-wall stress by Zymolase treatment, Pkc1-GFP proteins were re-localized similarly in both strains, forming between five to ten distinct foci at the cells' periphery (Fig. 7B). Together, these results suggest that Asc1p has no role in directing Pkc1p to its sites during the budding process, or in the re-localization of Pkc1p during cell-wall stress.

## Discussion

In mammalian cells RACK1 serves as a scaffold protein for numerous components of diverse signal transduction pathways, of which PKCβII is the most recognized [40]. In this study we show that in the budding yeast, loss of the RACK1 ortholog gene, *asc1*, results in phenotypes that characterize some PKC signaling mutants. In particular, *asc1*Δ strain was sensitive to staurosporine (Fig. 1), a specific inhibitor of Pkc1p in yeast [32], [33], and showed increased sensitivities to hypotonic- and heat shock conditions and to Zymolase treatment (Figs. 2, 3, 4 and 6D). These are known to provoke a cell-wall stress, which requires a functional PKC signaling for cells to survive.

The dynamics of *asc1*Δ loss of viability, whereby more than half of *asc1*Δ cells were damaged within the first minute of exposure to hypotonic conditions, is highly similar to the response of *pkc1*Δ strain (Fig. 3). However, unlike *pkc1*Δ strain that displayed unchanged to slow reduction in viability after the initial drop in survival, *asc1*Δ strain slowly resumed its growth. This may imply that *pkc1*Δ sensitivity to hypotonic conditions involves two distinct processes, of which *asc1*Δ susceptibility is linked only to the first, immediate one. Indeed, Levin *et al.* have shown that *PKC1*-deleted cells that possessed buds of any size underwent immediate lysis upon transfer to medium lacking osmotic stabilizers, while non budded cells arrested at the early stages of bud formation [35], [41]. Therefore, whereas Pkc1p function is important to all stages of bud formation Asc1p appears to have a role only in the mid-to late stages of this process.

Several observations in this work suggest that Asc1p is connected to Pkc1p by a different mechanism then in higher eukaryotes. First, in mammalian cells, RACK1 serves as a scaffold that mediates the phosphorylation and activation of the MAPK JNK by PKCβII [42]. However, our data suggest that *asc1*Δ cells contain high basal levels of PKC signaling (Fig. 5), which comes in agreement with the hyper-phsphorylation of the terminal MAPK, Slt2/Mpk1p, in *asc1*Δ background [25]. This observation raises the possibility that the PKC signaling is activated in *asc1*Δ strain to compensate for its cell-wall sensitivity due to loss of function of other mechanism. Second, in mammalian cells PKCβII-RACK1 interaction appears to target PKCβII to distinct intracellular locations, which vary between different cell types [6], [8], [10]. In yeast, full-length Pkc1p appears to reside predominantly at the bud-neck of medium to large sized buds and at the tip of small-sized buds, and to become re-localized to the cell's periphery upon exposure to cell-wall stress [29], [39]. Our results show that loss of Asc1p has no effect on Pkc1p localization at steady-state growth conditions nor on its re-distribution after cell-wall stress (Fig. 7B), and has no influence on Pkc1p fractionation with membrane-compartments (Fig. 7A). Third, while a physical interaction between Asc1 and Pkc1 proteins cannot be ruled out, we were unable to support these by two hybrid analyses or co-immunoprecipitation (data not shown). Moreover, the genetic interactions between the two genes suggest that they do not act in the same pathway. Specifically, double-knockout mutant lacking both *asc1* and *pkc1* genes displayed synergistic sensitivities to cell-wall stress conditions (Fig. 6C, D). Taken together, our observations suggest that in *S.cerevisiae*, Asc1p contribution to cell-wall integrity is not through the conventional Bck1-Mkk1/2-Mpk1 MAPK module that acts downstream to Pkc1p. Rather, Asc1p appears to function in parallel to Pkc1p, or to coordinate between the PKC signaling pathway and other cell-wall integrity related mechanisms, to the most.

How might Asc1p affect the integrity of the cell-wall? A likely mechanism is by regulating the translation of mRNAs that encode for cell-wall proteins. Indeed, mutations in Asc1p that were shown to reduce its ability to associate with ribosomes, resulted in also increased sensitivity to calcofluor white, a cell-wall perturbing agent [2]. Based on the immediate lysis of *asc1*Δ cells upon hypotonic shock conditions (Fig. 3) that was independent of de-novo synthesis of proteins (Fig. 4), we suggest that regulation of translation by Asc1 is exerted on bud-site related mRNAs during steady-state growth, rather than in response to cell-wall stress.
